# Surgical research and activity analysis using Hospital Episode Statistics

**DOI:** 10.1308/003588412X13373405385250

**Published:** 2012-05

**Authors:** JP Slavin, M Deakin, R Wilson

**Affiliations:** ^1^University of Keele,UK; ^2^West Midlands QI, NHS West Midlands,UK

**Keywords:** Episode of Care, Clinical Coding, Clinical Audit, Research

Hospital Episode Statistics (HES; http://www.hesonline.nhs.uk/) provide a national database for England that aims to capture all clinical activity in the National Health Service (NHS). The data are a valuable resource for surgical research and can be used for activity analysis, allowing comparisons of throughput and outcomes. HES are collated yearly by the Department of Health in England and contain information on every hospital attendance or admission. Data are collected as episodes. Each episode is a period of care in hospital under one consultant. Most patients only have one episode during an admission (or spell) but some will have multiple episodes and linking these to remove duplicates is often necessary. Although the data collected are for administrative purposes, information on patient diagnosis, operative procedure and outcome is available, making HES a valuable tool for research and audit.

The data are anonymised and this allows their widespread use without the need for patient consent. Requests for data that contain sensitive information (ie information that cannot identify a patient but is specific to a particular individual, such as date of birth) involve a more rigorous request process.

HESID is an anonymous and unique patient identifier derived from a patient’s NHS number, date of birth, sex and postcode.^1^ It allows the linking of different episodes that are from the same patient. These can be on the same admission or following further admissions to the same or another hospital. Essentially, the care of a patient may be followed across time and throughout England without breaking anonymity. HESID therefore provides a powerful method for assessing long-term outcome following surgical intervention. For example, readmissions at a later date with complications or sequelae of surgery can be picked up even if they occur at another hospital. Furthermore, HES can now be linked to the mortality data available from the Office for National Statistics, allowing deaths that occur outside hospital to be identified.^2^

Section 251 of the NHS Act 2006 (originally enacted under Section 60 of the Health and Social Care Act 2001) allows the common law duty of confidentiality to be set aside in specific circumstances where anonymised information is not sufficient and where patient consent is not practicable.^3^ In the case of a research study, this requires an application via the Integrated Research Application System or directly, in the case of service evaluation, to the National Information Governance Board for Health and Social Care. Using Section 251, it is possible to link HES using information that identifies a patient to other databases and to examine longer term outcome.

Details with regard to the consultant responsible for a case are also contained in the HES dataset. These are based on the consultant’s General Medical Council number, which is then anonymised. There are, however, no details on the operating surgeon and this remains a major omission.

Studies based on HES generate strong and polarised reactions among clinicians, mostly with regard to accuracy of coding. In fact, the reality is rather mixed.^4^ The accuracy of diagnostic and operative coding has improved over recent years, particularly for common operations as demonstrated in an annual national audit undertaken by the Department of Health and published for the first time in 2008.^5^ This audit did, however, show that there were significant coding irregularities, especially in certain trusts.

The coding issues themselves can be complex. There may be a coding error involving a diagnostic or a procedure code, or else administrative codes (eg the method of discharge or date of admission/discharge) may be incorrect. Often, however, the code that is used may be incorrect but only in a small way. For instance, the use of a code for laparoscopic cholecystectomy, when the code for laparoscopic cholecystectomy with on-table cholangiography should have been used. Moreover, depth of coding may vary. A patient admitted with a modest degree of ischaemia of a limb, for angiography, could conceivably be coded in exactly the same manner as a patient admitted with critical limb ischaemia with associated gangrene and sepsis. The coding is not incorrect but the lack of subsidiary codes fails to delineate the true clinical situation.

Although studies based on HES are cohort studies, they differ from the usual cohort studies in that the cohort represents almost all activity in the area of study in England as the contribution of the private sector is relatively small and NHS patients undergoing surgery in the private sector are included.

Furthermore, one has to consider the context of conclusions that are drawn from studies using HES. If findings are of a general nature (eg that patients in England with acute pancreatitis secondary to gallstones are not getting their cholecystectomy within the guidelines issued by the British Society of Gastroenterology), then even a relatively high coding error rate at some hospitals or even all hospitals will not detract markedly from the overall conclusions if significant deviation can be shown.^6^

However, if conclusions are of a much more specific nature (eg that hospital A is doing better than hospital B), then any conclusions drawn may well be incorrect unless it can be shown that the coding in hospital A is equivalent to the coding in hospital B. Thus, HES can usefully be used to test hypotheses nationally but at the current time must be used with caution when measuring individual hospital or clinician activity.^6^

This has not stopped the appearance of reports that evaluate clinical and surgical performance, for example the Dr Foster *Hospital Guide*.^7^ Many groups, including our own, are seeking to develop new metrics based on HES that can be usefully used to measure surgical care and outcome.

These are issues that need much greater discussion in the medical profession as it is unlikely that there will be any major changes in the method of diagnostic and operative coding. Although these are revised on a regular basis to reflect changing clinical practice, general agreement on standard ways of coding common procedures could be obtained easily through the specialty associations. Clinicians should understand and become engaged in the coding of their patients and the quality of the data that is returned on their behalf,^8^ not only to ensure good quality data for research but to safeguard the reputation of their hospital or surgical team.

## Corrigendum

The incidence of post operative venous thromboembolism in patients undergoing varicose vein surgery recorded in Hospital Episode Statistics. Sutton PA, El-Dhuwaib Y, Dyer J, Guy AJ. *Ann R Coll Surg Engl* 2011; **94**: 481-83

My El-Dhuwaib’s name was incorrectly spelt in the original publication.

We apologise for any confusion caused.
